# *N*-(6-Bromo­meth­yl-2-pyrid­yl)acetamide

**DOI:** 10.1107/S1600536810035026

**Published:** 2010-09-04

**Authors:** Hoong-Kun Fun, Jia Hao Goh, Nirmal Kumar Das, Debabrata Sen, Shyamaprosad Goswami

**Affiliations:** a X-ray Crystallography Unit, School of Physics, Universiti Sains Malaysia, 11800 USM, Penang, Malaysia; b Department of Chemistry, Bengal Engineering and Science University, Shibpur, Howrah 711 103, India

## Abstract

The title acetamide compound, C_8_H_9_BrN_2_O, crystallizes with three crystallographically independent mol­ecules (*A*, *B* and *C*) in the asymmetric unit. In mol­ecule *A*, the mean plane through the acetamide unit is inclined at a dihedral angle of 4.40 (11)° with respect to the pyridine ring [10.31 (12) and 2.27 (11)°, respectively, for mol­ecules *B* and *C*]. In the crystal structure, mol­ecules are inter­connected into sheets parallel to the *ac* plane by N—H⋯O, C—H⋯Br, C—H⋯O and C—H⋯N hydrogen bonds. The structure is further stabilized by weak inter­molecular C—H⋯π inter­actions.

## Related literature

For general background and applications of acetamide compounds, see: Goswami *et al.* (2000 ▶, 2005 ▶); Ghosh & Masanta (2006 ▶). For the preparation, see: Goswami *et al.* (2001 ▶, 2004 ▶). For the stability of the temperature controller used in the data collection, see: Cosier & Glazer (1986 ▶).
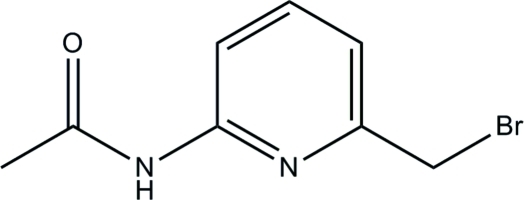


## Experimental

### 

#### Crystal data

C_8_H_9_BrN_2_O
*M* *_r_* = 229.08
Monoclinic, 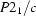

*a* = 4.1894 (8) Å
*b* = 26.219 (5) Å
*c* = 23.817 (4) Å
β = 94.148 (4)°
*V* = 2609.2 (8) Å^3^
*Z* = 12
Mo *K*α radiation
μ = 4.68 mm^−1^
*T* = 100 K
0.31 × 0.14 × 0.09 mm

#### Data collection

Bruker APEXII DUO CCD area-detector diffractometer
Absorption correction: multi-scan (*SADABS*; Bruker, 2009 ▶) *T*_min_ = 0.323, *T*_max_ = 0.668
72227 measured reflections
10228 independent reflections
8239 reflections with *I* > 2σ(*I*)
*R*_int_ = 0.058

#### Refinement

*R*[*F* ^2^ > 2σ(*F* ^2^)] = 0.033
*wR*(*F* ^2^) = 0.100
*S* = 1.06
10228 reflections
340 parameters
H atoms treated by a mixture of independent and constrained refinement
Δρ_max_ = 1.37 e Å^−3^
Δρ_min_ = −0.74 e Å^−3^

### 

Data collection: *APEX2* (Bruker, 2009 ▶); cell refinement: *SAINT* (Bruker, 2009 ▶); data reduction: *SAINT*; program(s) used to solve structure: *SHELXTL* (Sheldrick, 2008 ▶); program(s) used to refine structure: *SHELXTL*; molecular graphics: *SHELXTL* software used to prepare material for publication: *SHELXTL* and *PLATON* (Spek, 2009 ▶).

## Supplementary Material

Crystal structure: contains datablocks global, I. DOI: 10.1107/S1600536810035026/ci5177sup1.cif

Structure factors: contains datablocks I. DOI: 10.1107/S1600536810035026/ci5177Isup2.hkl

Additional supplementary materials:  crystallographic information; 3D view; checkCIF report

## Figures and Tables

**Table 1:** Hydrogen-bond geometry (Å, °) *Cg*1 and *Cg*2 are the centroids of the C2*A*–C6*A*/N1*A* and C2*C*–C6*C*/N1*C* pyridine rings, respectively.

*D*—H⋯*A*	*D*—H	H⋯*A*	*D*⋯*A*	*D*—H⋯*A*
N2*A*—H2*NA*⋯O1*C*^i^	0.74 (3)	2.29 (3)	3.022 (2)	172 (4)
N2*B*—H2*NB*⋯O1*A*	0.93 (3)	1.97 (3)	2.885 (2)	166 (3)
N2*C*—H2*NC*⋯O1*B*^ii^	0.73 (3)	2.18 (3)	2.900 (2)	169 (3)
C1*B*—H1*BA*⋯Br1*B*^iii^	0.97	2.85	3.716 (2)	149
C8*B*—H8*BB*⋯O1*A*	0.96	2.50	3.159 (3)	125
C8*C*—H8*CA*⋯N1*A*^iv^	0.96	2.50	3.427 (3)	162
C1*A*—H1*AB*⋯*Cg*1^iii^	0.97	2.88	3.612 (2)	133
C1*C*—H1*CB*⋯*Cg*2^iii^	0.97	2.81	3.447 (2)	124

Symmetry codes: (i) 
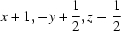
; (ii) 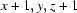; (iii) 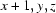; (iv) 
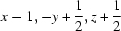
.

**Fig. 1.**
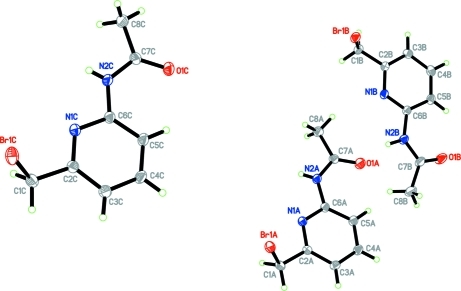
The molecular structure of the title compound, showing 50% probability displacement ellipsoids for non-H atoms and the atom-numbering scheme.

**Fig. 2.**
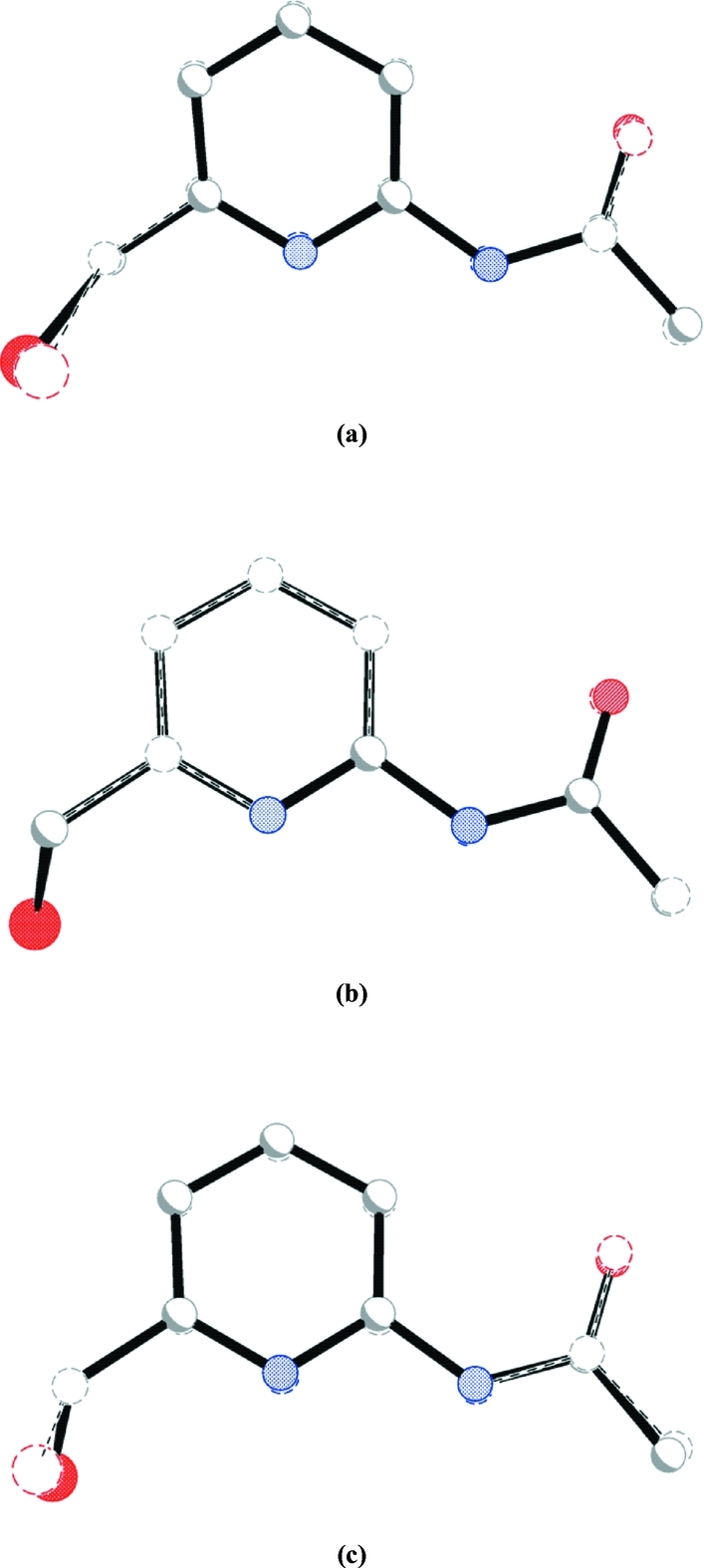
Fit of (a) molecule A (dashed lines) on molecule B (solid lines), (b) molecule C (dashed lines) on molecule A (solid lines), (c) molecule C (dashed lines) on molecule B (solid lines). H atoms have been omitted for clarity.

**Fig. 3.**
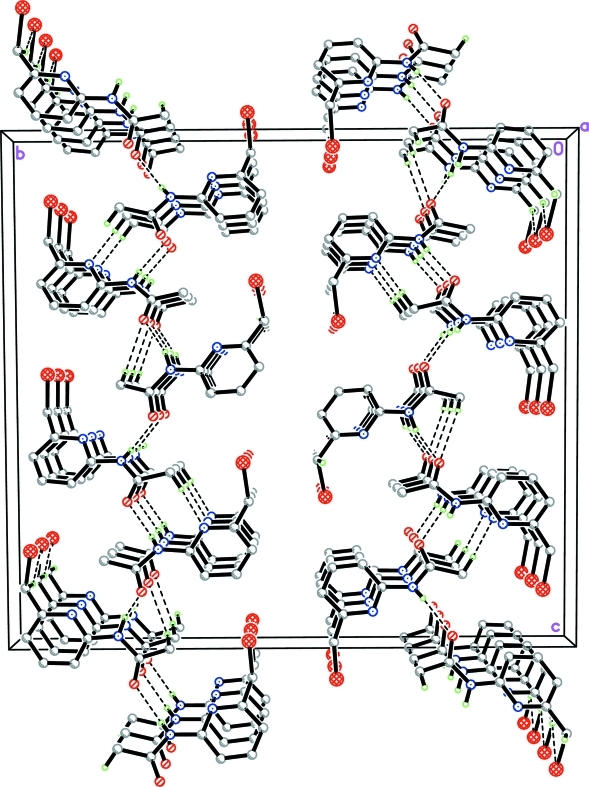
The crystal structure of the title compound, viewed along the a axis. Intermolecular hydrogen bonds are shown as dashed lines.

## supplementary crystallographic information

### Comment

Pyridine amides having bromine in side chains are enormously useful as they are suitable intermediates for the synthesis of flexible receptors for various biologically important substrates. In addition, they can easily be coupled with alcohol by Williamson reaction and to the amine by a simple reaction with a base. These types of compounds are therefore attracting the attention of molecular recognition chemist (Goswami *et al.*, 2000, 2005; Ghosh & Masanta, 2006).

The title acetamide compound crystallizes in space group *P*2_1_/*c* with three crystallographically independent molecules in the asymmetric unit, designated *A*, *B* and *C* (Fig. 1). The molecular geometries of all molecules are essentially similar, as indicated by the r.m.s. deviations for the superposition of the non-H atoms of any pair of molecules using *XP* in *SHELXTL* (Sheldrick, 2008) being 0.137 (*A*/*B* pair), 0.026 (*A*/*C* pair) and 0.130 Å (*B*/*C* pair). The superposition of molecular pairs are shown in Fig. 2. The corresponding geometric parameters of the three molecules agree well with each other. In molecule *A*, the mean plane formed through the acetamide moiety (N2A/C7A/C8A/O1A) is inclined at an interplanar angle of 4.40 (11)° with the pyridine ring (C2A-C6A/N1A); the respective angles for molecules *B* and *C* are 10.31 (2) and 2.27 (11)°, respectively.

In the crystal structure, intermolecular N2A—H2NA···O1C, N2B—H2NB···O1A, N2C—H2NC···O1B, C1B—H1BA···Br1B, C8B—H8BB···O1A and C8C—H8CA···N1A hydrogen bonds (Table 1) interconnect molecules into two-molecule-wide arrays parallel to *ac* plane (Fig. 3). Further stabilization of the crystal structure is provided by weak intermolecular C1A—H1AB···*Cg*1 and C1C—H1CB···*Cg2* interactions (Table 1) where *Cg*1 and *Cg*2 are the centroids of C2A-C6A/N1A and C2C-C6C/N1C pyridine rings, respectively.

### Experimental

The title compound was prepared according to literature procedures (Goswami *et al.*, 2001, 2004) and was recrystallized from a mixture of CHCl_3_ and CH_3_OH (9:1) by slow evaporation method.

### Refinement

H atoms bound to N atoms are located in a difference Fourier map and allowed to refine freely [range of N—H = 0.73 (3)–0.93 (3) Å]. The remaining H atoms were placed in their calculated positions, with C—H = 0.93–0.97 Å, and refined using a riding model, with *U*_iso_ = 1.2 or 1.5 *U*_eq_(C). The rotating group model was applied to methyl groups.

### Crystal data

**Table:**

C_8_H_9_BrN_2_O	*F*(000) = 1368
*M**_r_* = 229.08	*D*_x_ = 1.750 Mg m^−^^3^
Monoclinic, *P*2_1_/*c*	Mo *K*α radiation, λ = 0.71073 Å
Hall symbol: -P 2ybc	Cell parameters from 9903 reflections
*a* = 4.1894 (8) Å	θ = 3.0–33.5°
*b* = 26.219 (5) Å	µ = 4.68 mm^−^^1^
*c* = 23.817 (4) Å	*T* = 100 K
β = 94.148 (4)°	Plate, brown
*V* = 2609.2 (8) Å^3^	0.31 × 0.14 × 0.09 mm
*Z* = 12	

### Data collection

**Table:**

Bruker APEXII DUO CCD area-detector diffractometer	10228 independent reflections
Radiation source: fine-focus sealed tube	8239 reflections with *I* > 2σ(*I*)
graphite	*R*_int_ = 0.058
φ and ω scans	θ_max_ = 33.7°, θ_min_ = 1.8°
Absorption correction: multi-scan (*SADABS*; Bruker, 2009)	*h* = −6→6
*T*_min_ = 0.323, *T*_max_ = 0.668	*k* = −40→40
72227 measured reflections	*l* = −36→36

### Refinement

**Table:**

Refinement on *F*^2^	Primary atom site location: structure-invariant direct methods
Least-squares matrix: full	Secondary atom site location: difference Fourier map
*R*[*F*^2^ > 2σ(*F*^2^)] = 0.033	Hydrogen site location: inferred from neighbouring sites
*wR*(*F*^2^) = 0.100	H atoms treated by a mixture of independent and constrained refinement
*S* = 1.06	*w* = 1/[σ^2^(*F*_o_^2^) + (0.052*P*)^2^ + 0.7624*P*] where *P* = (*F*_o_^2^ + 2*F*_c_^2^)/3
10228 reflections	(Δ/σ)_max_ = 0.005
340 parameters	Δρ_max_ = 1.37 e Å^−^^3^
0 restraints	Δρ_min_ = −0.74 e Å^−^^3^

### Special details

**Table:**

Experimental. The crystal was placed in the cold stream of an Oxford Cryosystems Cobra open-flow nitrogen cryostat (Cosier & Glazer, 1986) operating at 100.0 (1)K.
Geometry. All esds (except the esd in the dihedral angle between two l.s. planes) are estimated using the full covariance matrix. The cell esds are taken into account individually in the estimation of esds in distances, angles and torsion angles; correlations between esds in cell parameters are only used when they are defined by crystal symmetry. An approximate (isotropic) treatment of cell esds is used for estimating esds involving l.s. planes.
Refinement. Refinement of F^2^ against ALL reflections. The weighted R-factor wR and goodness of fit S are based on F^2^, conventional R-factors R are based on F, with F set to zero for negative F^2^. The threshold expression of F^2^ > 2sigma(F^2^) is used only for calculating R-factors(gt) etc. and is not relevant to the choice of reflections for refinement. R-factors based on F^2^ are statistically about twice as large as those based on F, and R- factors based on ALL data will be even larger.

### Fractional atomic coordinates and isotropic or equivalent isotropic displacement parameters (Å^2^)

**Table:**

	*x*	*y*	*z*	*U*_iso_*/*U*_eq_	
Br1A	1.14992 (5)	0.418118 (8)	0.367324 (7)	0.02031 (5)	
O1A	0.5948 (4)	0.25161 (6)	0.13677 (6)	0.0296 (3)	
N1A	1.1052 (4)	0.35205 (6)	0.24649 (6)	0.0167 (3)	
N2A	0.9447 (4)	0.27575 (6)	0.20938 (7)	0.0191 (3)	
C1A	1.3223 (5)	0.42675 (8)	0.29327 (7)	0.0196 (3)	
H1AA	1.3460	0.4628	0.2856	0.024*	
H1AB	1.5326	0.4112	0.2939	0.024*	
C2A	1.1091 (4)	0.40309 (7)	0.24723 (7)	0.0166 (3)	
C3A	0.9351 (5)	0.43276 (7)	0.20772 (7)	0.0186 (3)	
H3AA	0.9413	0.4682	0.2095	0.022*	
C4A	0.7516 (5)	0.40783 (7)	0.16540 (7)	0.0200 (3)	
H4AA	0.6331	0.4267	0.1382	0.024*	
C5A	0.7432 (5)	0.35522 (7)	0.16334 (7)	0.0195 (3)	
H5AA	0.6212	0.3381	0.1351	0.023*	
C6A	0.9266 (4)	0.32861 (7)	0.20556 (7)	0.0171 (3)	
C7A	0.7892 (5)	0.24022 (7)	0.17560 (8)	0.0198 (3)	
C8A	0.8755 (5)	0.18609 (7)	0.18922 (8)	0.0235 (4)	
H8AA	0.7098	0.1639	0.1735	0.035*	
H8AB	0.8985	0.1818	0.2293	0.035*	
H8AC	1.0738	0.1778	0.1735	0.035*	
Br1B	0.14297 (5)	0.058241 (8)	0.203481 (7)	0.02180 (5)	
O1B	0.0701 (5)	0.23172 (6)	−0.04493 (7)	0.0359 (4)	
N1B	0.2651 (4)	0.12464 (6)	0.08249 (6)	0.0174 (3)	
N2B	0.2563 (4)	0.20320 (6)	0.04135 (7)	0.0206 (3)	
C1B	0.3307 (5)	0.04503 (8)	0.13130 (8)	0.0209 (3)	
H1BA	0.5573	0.0530	0.1351	0.025*	
H1BB	0.3086	0.0091	0.1221	0.025*	
C2B	0.1736 (4)	0.07590 (7)	0.08467 (7)	0.0170 (3)	
C3B	−0.0419 (5)	0.05349 (7)	0.04525 (8)	0.0199 (3)	
H3BA	−0.1004	0.0194	0.0482	0.024*	
C4B	−0.1672 (5)	0.08373 (8)	0.00115 (8)	0.0215 (4)	
H4BA	−0.3132	0.0700	−0.0260	0.026*	
C5B	−0.0763 (5)	0.13402 (8)	−0.00257 (8)	0.0209 (3)	
H5BA	−0.1573	0.1546	−0.0321	0.025*	
C6B	0.1427 (5)	0.15320 (7)	0.03964 (7)	0.0174 (3)	
C7B	0.2223 (6)	0.23918 (8)	0.00022 (8)	0.0251 (4)	
C8B	0.3835 (7)	0.28927 (9)	0.01365 (10)	0.0352 (5)	
H8BA	0.4373	0.3054	−0.0206	0.053*	
H8BB	0.5750	0.2836	0.0375	0.053*	
H8BC	0.2414	0.3109	0.0327	0.053*	
Br1C	1.07629 (5)	0.426384 (8)	1.034127 (8)	0.02481 (6)	
O1C	0.3915 (4)	0.28054 (6)	0.79326 (6)	0.0243 (3)	
N1C	0.9830 (4)	0.36463 (6)	0.90997 (6)	0.0173 (3)	
N2C	0.7488 (4)	0.29499 (6)	0.86856 (7)	0.0201 (3)	
C1C	1.2666 (5)	0.43125 (8)	0.96116 (8)	0.0218 (3)	
H1CA	1.3315	0.4662	0.9550	0.026*	
H1CB	1.4563	0.4100	0.9619	0.026*	
C2C	1.0370 (5)	0.41486 (7)	0.91382 (7)	0.0174 (3)	
C3C	0.8994 (5)	0.44982 (7)	0.87597 (7)	0.0202 (3)	
H3CA	0.9409	0.4845	0.8800	0.024*	
C4C	0.6977 (5)	0.43147 (8)	0.83191 (7)	0.0209 (3)	
H4CA	0.6031	0.4541	0.8056	0.025*	
C5C	0.6360 (5)	0.37991 (7)	0.82678 (7)	0.0194 (3)	
H5CA	0.5004	0.3671	0.7975	0.023*	
C6C	0.7858 (4)	0.34775 (7)	0.86741 (7)	0.0168 (3)	
C7C	0.5659 (5)	0.26431 (7)	0.83320 (7)	0.0196 (3)	
C8C	0.5926 (6)	0.20884 (8)	0.84701 (9)	0.0271 (4)	
H8CA	0.5007	0.1892	0.8159	0.041*	
H8CB	0.4802	0.2018	0.8799	0.041*	
H8CC	0.8140	0.1999	0.8541	0.041*	
H2NA	1.062 (8)	0.2645 (13)	0.2303 (13)	0.045 (9)*	
H2NB	0.372 (8)	0.2135 (12)	0.0743 (12)	0.039 (8)*	
H2NC	0.850 (8)	0.2790 (12)	0.8879 (12)	0.033 (8)*	

### Atomic displacement parameters (Å^2^)

**Table:**

	*U*^11^	*U*^22^	*U*^33^	*U*^12^	*U*^13^	*U*^23^
Br1A	0.02104 (9)	0.02320 (9)	0.01647 (8)	−0.00081 (6)	−0.00005 (6)	−0.00327 (6)
O1A	0.0389 (9)	0.0208 (7)	0.0268 (7)	−0.0026 (6)	−0.0141 (6)	−0.0012 (6)
N1A	0.0182 (7)	0.0163 (7)	0.0152 (6)	−0.0005 (5)	−0.0012 (5)	−0.0013 (5)
N2A	0.0228 (8)	0.0159 (7)	0.0177 (7)	0.0005 (6)	−0.0055 (6)	−0.0011 (5)
C1A	0.0198 (8)	0.0209 (8)	0.0181 (7)	−0.0055 (6)	0.0007 (6)	−0.0008 (6)
C2A	0.0166 (8)	0.0171 (7)	0.0163 (7)	−0.0012 (6)	0.0014 (6)	−0.0006 (6)
C3A	0.0233 (9)	0.0161 (7)	0.0163 (7)	0.0004 (6)	0.0015 (6)	0.0022 (6)
C4A	0.0235 (9)	0.0201 (8)	0.0163 (7)	0.0014 (7)	−0.0007 (6)	0.0018 (6)
C5A	0.0238 (9)	0.0194 (8)	0.0146 (7)	0.0004 (6)	−0.0032 (6)	−0.0006 (6)
C6A	0.0187 (8)	0.0175 (8)	0.0148 (7)	0.0001 (6)	−0.0005 (6)	−0.0019 (6)
C7A	0.0234 (9)	0.0171 (8)	0.0186 (7)	−0.0017 (6)	−0.0007 (6)	−0.0022 (6)
C8A	0.0287 (10)	0.0177 (8)	0.0236 (8)	−0.0001 (7)	−0.0020 (7)	−0.0032 (7)
Br1B	0.02550 (10)	0.02243 (10)	0.01691 (8)	0.00067 (7)	−0.00224 (6)	0.00250 (6)
O1B	0.0555 (11)	0.0220 (7)	0.0271 (7)	−0.0039 (7)	−0.0182 (7)	0.0056 (6)
N1B	0.0190 (7)	0.0172 (7)	0.0156 (6)	0.0021 (5)	−0.0022 (5)	−0.0002 (5)
N2B	0.0279 (8)	0.0166 (7)	0.0163 (6)	0.0006 (6)	−0.0051 (6)	−0.0002 (5)
C1B	0.0214 (9)	0.0194 (8)	0.0218 (8)	0.0032 (6)	0.0007 (7)	0.0027 (6)
C2B	0.0175 (8)	0.0177 (8)	0.0157 (7)	0.0022 (6)	0.0008 (6)	0.0002 (6)
C3B	0.0219 (9)	0.0194 (8)	0.0186 (7)	−0.0021 (7)	0.0020 (6)	−0.0033 (6)
C4B	0.0225 (9)	0.0252 (9)	0.0165 (7)	−0.0013 (7)	−0.0018 (6)	−0.0035 (6)
C5B	0.0224 (9)	0.0234 (9)	0.0161 (7)	0.0021 (7)	−0.0032 (6)	−0.0008 (6)
C6B	0.0202 (8)	0.0165 (7)	0.0154 (7)	0.0015 (6)	−0.0005 (6)	−0.0010 (6)
C7B	0.0341 (11)	0.0175 (8)	0.0224 (8)	0.0024 (7)	−0.0064 (7)	0.0018 (7)
C8B	0.0512 (15)	0.0194 (9)	0.0323 (11)	−0.0037 (9)	−0.0145 (10)	0.0049 (8)
Br1C	0.02540 (10)	0.03212 (11)	0.01646 (8)	0.00276 (7)	−0.00162 (7)	−0.00555 (7)
O1C	0.0274 (8)	0.0223 (7)	0.0217 (6)	−0.0020 (5)	−0.0090 (5)	0.0002 (5)
N1C	0.0183 (7)	0.0182 (7)	0.0150 (6)	−0.0008 (5)	−0.0012 (5)	−0.0007 (5)
N2C	0.0254 (8)	0.0161 (7)	0.0175 (7)	−0.0022 (6)	−0.0071 (6)	0.0018 (5)
C1C	0.0219 (9)	0.0223 (8)	0.0210 (8)	−0.0039 (7)	−0.0010 (6)	−0.0019 (7)
C2C	0.0189 (8)	0.0189 (8)	0.0145 (7)	−0.0011 (6)	0.0013 (6)	−0.0009 (6)
C3C	0.0260 (9)	0.0181 (8)	0.0162 (7)	−0.0017 (7)	−0.0004 (6)	−0.0011 (6)
C4C	0.0262 (9)	0.0208 (8)	0.0152 (7)	0.0015 (7)	−0.0025 (6)	0.0032 (6)
C5C	0.0221 (9)	0.0203 (8)	0.0151 (7)	0.0008 (6)	−0.0031 (6)	0.0006 (6)
C6C	0.0186 (8)	0.0174 (8)	0.0142 (7)	0.0000 (6)	−0.0009 (6)	−0.0006 (6)
C7C	0.0210 (9)	0.0198 (8)	0.0177 (7)	−0.0025 (6)	−0.0012 (6)	−0.0019 (6)
C8C	0.0355 (12)	0.0187 (9)	0.0254 (9)	−0.0045 (8)	−0.0099 (8)	0.0008 (7)

### Geometric parameters (Å, °)

**Table:**

Br1A—C1A	1.9667 (18)	C3B—C4B	1.389 (3)
O1A—C7A	1.224 (2)	C3B—H3BA	0.93
N1A—C6A	1.335 (2)	C4B—C5B	1.377 (3)
N1A—C2A	1.338 (2)	C4B—H4BA	0.93
N2A—C7A	1.365 (2)	C5B—C6B	1.404 (3)
N2A—C6A	1.391 (2)	C5B—H5BA	0.93
N2A—H2NA	0.74 (3)	C7B—C8B	1.501 (3)
C1A—C2A	1.498 (3)	C8B—H8BA	0.96
C1A—H1AA	0.97	C8B—H8BB	0.96
C1A—H1AB	0.97	C8B—H8BC	0.96
C2A—C3A	1.387 (3)	Br1C—C1C	1.968 (2)
C3A—C4A	1.386 (3)	O1C—C7C	1.233 (2)
C3A—H3AA	0.93	N1C—C6C	1.336 (2)
C4A—C5A	1.380 (3)	N1C—C2C	1.338 (2)
C4A—H4AA	0.93	N2C—C7C	1.360 (2)
C5A—C6A	1.406 (2)	N2C—C6C	1.392 (2)
C5A—H5AA	0.93	N2C—H2NC	0.73 (3)
C7A—C8A	1.494 (3)	C1C—C2C	1.491 (3)
C8A—H8AA	0.96	C1C—H1CA	0.97
C8A—H8AB	0.96	C1C—H1CB	0.97
C8A—H8AC	0.96	C2C—C3C	1.382 (3)
Br1B—C1B	1.9722 (19)	C3C—C4C	1.385 (3)
O1B—C7B	1.225 (2)	C3C—H3CA	0.93
N1B—C2B	1.336 (2)	C4C—C5C	1.380 (3)
N1B—C6B	1.338 (2)	C4C—H4CA	0.93
N2B—C7B	1.360 (2)	C5C—C6C	1.398 (2)
N2B—C6B	1.394 (2)	C5C—H5CA	0.93
N2B—H2NB	0.93 (3)	C7C—C8C	1.494 (3)
C1B—C2B	1.489 (3)	C8C—H8CA	0.96
C1B—H1BA	0.97	C8C—H8CB	0.96
C1B—H1BB	0.97	C8C—H8CC	0.96
C2B—C3B	1.385 (3)		
			
C6A—N1A—C2A	118.40 (15)	C5B—C4B—H4BA	119.9
C7A—N2A—C6A	128.33 (16)	C3B—C4B—H4BA	119.9
C7A—N2A—H2NA	113 (3)	C4B—C5B—C6B	117.78 (17)
C6A—N2A—H2NA	118 (3)	C4B—C5B—H5BA	121.1
C2A—C1A—Br1A	111.74 (13)	C6B—C5B—H5BA	121.1
C2A—C1A—H1AA	109.3	N1B—C6B—N2B	113.14 (16)
Br1A—C1A—H1AA	109.3	N1B—C6B—C5B	122.70 (17)
C2A—C1A—H1AB	109.3	N2B—C6B—C5B	124.16 (16)
Br1A—C1A—H1AB	109.3	O1B—C7B—N2B	122.83 (19)
H1AA—C1A—H1AB	107.9	O1B—C7B—C8B	121.62 (19)
N1A—C2A—C3A	123.14 (17)	N2B—C7B—C8B	115.54 (17)
N1A—C2A—C1A	115.46 (16)	C7B—C8B—H8BA	109.5
C3A—C2A—C1A	121.39 (17)	C7B—C8B—H8BB	109.5
C4A—C3A—C2A	117.74 (17)	H8BA—C8B—H8BB	109.5
C4A—C3A—H3AA	121.1	C7B—C8B—H8BC	109.5
C2A—C3A—H3AA	121.1	H8BA—C8B—H8BC	109.5
C5A—C4A—C3A	120.55 (17)	H8BB—C8B—H8BC	109.5
C5A—C4A—H4AA	119.7	C6C—N1C—C2C	118.06 (16)
C3A—C4A—H4AA	119.7	C7C—N2C—C6C	129.32 (16)
C4A—C5A—C6A	117.35 (17)	C7C—N2C—H2NC	109 (2)
C4A—C5A—H5AA	121.3	C6C—N2C—H2NC	121 (2)
C6A—C5A—H5AA	121.3	C2C—C1C—Br1C	111.62 (13)
N1A—C6A—N2A	112.72 (16)	C2C—C1C—H1CA	109.3
N1A—C6A—C5A	122.82 (17)	Br1C—C1C—H1CA	109.3
N2A—C6A—C5A	124.46 (16)	C2C—C1C—H1CB	109.3
O1A—C7A—N2A	122.80 (18)	Br1C—C1C—H1CB	109.3
O1A—C7A—C8A	122.20 (17)	H1CA—C1C—H1CB	108.0
N2A—C7A—C8A	114.99 (17)	N1C—C2C—C3C	123.20 (17)
C7A—C8A—H8AA	109.5	N1C—C2C—C1C	115.60 (16)
C7A—C8A—H8AB	109.5	C3C—C2C—C1C	121.18 (17)
H8AA—C8A—H8AB	109.5	C2C—C3C—C4C	117.81 (18)
C7A—C8A—H8AC	109.5	C2C—C3C—H3CA	121.1
H8AA—C8A—H8AC	109.5	C4C—C3C—H3CA	121.1
H8AB—C8A—H8AC	109.5	C5C—C4C—C3C	120.48 (17)
C2B—N1B—C6B	118.02 (16)	C5C—C4C—H4CA	119.8
C7B—N2B—C6B	127.83 (16)	C3C—C4C—H4CA	119.8
C7B—N2B—H2NB	115.3 (19)	C4C—C5C—C6C	117.26 (17)
C6B—N2B—H2NB	116.8 (19)	C4C—C5C—H5CA	121.4
C2B—C1B—Br1B	111.85 (13)	C6C—C5C—H5CA	121.4
C2B—C1B—H1BA	109.2	N1C—C6C—N2C	112.20 (16)
Br1B—C1B—H1BA	109.2	N1C—C6C—C5C	123.19 (17)
C2B—C1B—H1BB	109.2	N2C—C6C—C5C	124.62 (16)
Br1B—C1B—H1BB	109.2	O1C—C7C—N2C	123.35 (18)
H1BA—C1B—H1BB	107.9	O1C—C7C—C8C	122.47 (17)
N1B—C2B—C3B	123.66 (17)	N2C—C7C—C8C	114.18 (16)
N1B—C2B—C1B	115.86 (16)	C7C—C8C—H8CA	109.5
C3B—C2B—C1B	120.41 (17)	C7C—C8C—H8CB	109.5
C2B—C3B—C4B	117.55 (18)	H8CA—C8C—H8CB	109.5
C2B—C3B—H3BA	121.2	C7C—C8C—H8CC	109.5
C4B—C3B—H3BA	121.2	H8CA—C8C—H8CC	109.5
C5B—C4B—C3B	120.29 (18)	H8CB—C8C—H8CC	109.5
			
C6A—N1A—C2A—C3A	0.4 (3)	C2B—N1B—C6B—N2B	−179.55 (16)
C6A—N1A—C2A—C1A	−178.19 (16)	C2B—N1B—C6B—C5B	0.1 (3)
Br1A—C1A—C2A—N1A	−71.41 (19)	C7B—N2B—C6B—N1B	168.3 (2)
Br1A—C1A—C2A—C3A	110.00 (18)	C7B—N2B—C6B—C5B	−11.3 (3)
N1A—C2A—C3A—C4A	−0.5 (3)	C4B—C5B—C6B—N1B	0.3 (3)
C1A—C2A—C3A—C4A	177.93 (17)	C4B—C5B—C6B—N2B	179.93 (19)
C2A—C3A—C4A—C5A	0.3 (3)	C6B—N2B—C7B—O1B	2.6 (4)
C3A—C4A—C5A—C6A	0.1 (3)	C6B—N2B—C7B—C8B	−177.6 (2)
C2A—N1A—C6A—N2A	179.68 (16)	C6C—N1C—C2C—C3C	−0.1 (3)
C2A—N1A—C6A—C5A	0.0 (3)	C6C—N1C—C2C—C1C	−178.23 (17)
C7A—N2A—C6A—N1A	178.45 (19)	Br1C—C1C—C2C—N1C	−73.37 (19)
C7A—N2A—C6A—C5A	−1.9 (3)	Br1C—C1C—C2C—C3C	108.44 (18)
C4A—C5A—C6A—N1A	−0.2 (3)	N1C—C2C—C3C—C4C	−0.4 (3)
C4A—C5A—C6A—N2A	−179.84 (18)	C1C—C2C—C3C—C4C	177.65 (18)
C6A—N2A—C7A—O1A	−2.9 (3)	C2C—C3C—C4C—C5C	0.6 (3)
C6A—N2A—C7A—C8A	176.72 (18)	C3C—C4C—C5C—C6C	−0.3 (3)
C6B—N1B—C2B—C3B	−0.4 (3)	C2C—N1C—C6C—N2C	−179.58 (17)
C6B—N1B—C2B—C1B	176.64 (16)	C2C—N1C—C6C—C5C	0.4 (3)
Br1B—C1B—C2B—N1B	78.77 (19)	C7C—N2C—C6C—N1C	178.90 (19)
Br1B—C1B—C2B—C3B	−104.14 (18)	C7C—N2C—C6C—C5C	−1.1 (3)
N1B—C2B—C3B—C4B	0.2 (3)	C4C—C5C—C6C—N1C	−0.2 (3)
C1B—C2B—C3B—C4B	−176.68 (17)	C4C—C5C—C6C—N2C	179.76 (19)
C2B—C3B—C4B—C5B	0.3 (3)	C6C—N2C—C7C—O1C	−1.6 (3)
C3B—C4B—C5B—C6B	−0.5 (3)	C6C—N2C—C7C—C8C	178.7 (2)

### Hydrogen-bond geometry (Å, °)

**Table:**

Cg1 and Cg2 are the centroids of the C2A–C6A/N1A and C2C–C6C/N1C pyridine rings, respectively.

**Table:**

*D*—H···*A*	*D*—H	H···*A*	*D*···*A*	*D*—H···*A*
N2A—H2NA···O1C^i^	0.74 (3)	2.29 (3)	3.022 (2)	172 (4)
N2B—H2NB···O1A	0.93 (3)	1.97 (3)	2.885 (2)	166 (3)
N2C—H2NC···O1B^ii^	0.73 (3)	2.18 (3)	2.900 (2)	169 (3)
C1B—H1BA···Br1B^iii^	0.97	2.85	3.716 (2)	149
C8B—H8BB···O1A	0.96	2.50	3.159 (3)	125
C8C—H8CA···N1A^iv^	0.96	2.50	3.427 (3)	162
C1A—H1AB···Cg1^iii^	0.97	2.88	3.612 (2)	133
C1C—H1CB···Cg2^iii^	0.97	2.81	3.447 (2)	124

Symmetry codes: (i) *x*+1, −*y*+1/2, *z*−1/2; (ii) *x*+1, *y*, *z*+1; (iii) *x*+1, *y*, *z*; (iv) *x*−1, −*y*+1/2, *z*+1/2.
